# Does the mouth seed the small bowel? Mechanisms and hypothesis-generating framework via the oral–gut axis

**DOI:** 10.1080/20002297.2026.2735085

**Published:** 2026-10-28

**Authors:** Lin He, Jing Wang, Tao Bai, Xiaohua Hou, Di Ran, Jie Yang

**Affiliations:** a Department of Gastroenterology, the First Affiliated Hospital of Guizhou Medical University, Guiyang City, Guizhou Province, People's Republic of China; b Division of Gastroenterology, Union Hospital, Tongji Medical College, Huazhong University of Science and Technology, Wuhan City, Hubei Province, People's Republic of China

**Keywords:** Oral hygiene, oral microbiome, small intestinal bacterial overgrowth, oral–gut axis, microbial migration

## Abstract

**Background:**

As the starting segment of the digestive tract, the oral cavity harbors a complex microbial community that closely interacts with the distal gut microbiota. Recent evidence suggests that oral hygiene status and oral dysbiosis may correlate with gut microbial homeostasis and play a potential role in the initiation or exacerbation of small intestinal bacterial overgrowth (SIBO).

**Objective:**

This review aims to integrate existing evidence, outline a hypothesis-generating framework, identify critical knowledge gaps, and provide new perspectives for future interdisciplinary mechanistic research and clinical translation in SIBO and related gastrointestinal disorders.

**Design:**

This narrative review synthesizes and evaluates preliminary clinical correlations, metagenomic surveys, and animal models that support the oral microbiota as a potentially modifiable factor associated with SIBO, and discusses the proposed underlying molecular pathways.

**Results:**

Existing evidence supports the oral microbiota as a potentially modifiable factor associated with SIBO. However, direct high-level evidence testing oral hygiene interventions against definitive SIBO outcomes is currently lacking.

**Conclusions:**

The oral-gut axis may play a critical role in SIBO pathogenesis. Future research requires high-quality clinical trials to validate the impact of oral hygiene interventions on SIBO outcomes, bridging the current critical knowledge gaps.

## Key messages

- This review synthesizes available evidence to propose a hypothesis-generating framework in which oral microbial dysbiosis may be related to small intestinal bacterial overgrowth (SIBO) via the oral–gut axis.
- The core proposition is that targeted oral hygiene practices may theoretically reduce the descending load of oral “disruptor taxa” into the small intestine, thereby potentially lowering SIBO risk or recurrence, and it outlines possible mechanisms. It clearly points out that there is currently a lack of high-quality evidence on the effects of oral hygiene interventions on SIBO outcomes, and prospective validation is needed.

## Introduction

Within the complex human microecosystem, the oral cavity and the large intestine represent two major microbial reservoirs of the digestive tract, with the colon maintaining the absolute highest microbial colonisation density in the human body [1]. Advances in high‑throughput sequencing and the rapid development of metagenomics have gradually revealed a close functional connection between the oral cavity and the gut. The two sites are not independent; rather, they are tightly linked through physiological pathways, together forming a microbe–immune regulatory network that influences distal organs, including the gut [2–5].

In recent years, the concept of the oral–gut axis has emerged to describe the bidirectional, dynamic interaction between the oral and intestinal microbial ecosystems [6]. This concept has fundamentally updated our traditional understanding of the relationship between local infections and systemic diseases. Accumulating evidence indicates that orally derived microorganisms and their metabolites can migrate along the oral–gut axis, correlating with downstream gut dysbiosis, potentially influencing aberrant immune responses, and interacting with intestinal barrier integrity, leading to “leaky gut” conditions [7]. These mechanisms are frequently associated with the development of various systemic conditions, including inflammatory bowel disease, colorectal cancer, metabolic syndrome, rheumatoid arthritis, and Alzheimer’s disease [8–11]. Studies further suggest that the oral cavity can serve as a crucial gateway for microbial translocation through gingival leakage, profoundly affecting gut function and systemic health [12].

Among the various manifestations of gut dysbiosis, small intestinal bacterial overgrowth (SIBO) has become a particularly challenging clinical syndrome in gastroenterology due to its complex pathophysiology and diverse clinical presentations [13]. SIBO is characterised by an abnormal quantity or composition of bacteria in the small intestine, which disrupts normal digestive and absorptive functions, leading to symptoms such as bloating, abdominal pain, diarrhoea, constipation, and malnutrition [13]. It also shows high comorbidity with several functional gastrointestinal disorders, including irritable bowel syndrome [13] and functional dyspepsia [14]. Nevertheless, a fundamental question remains largely unanswered: where do the abnormally proliferating bacteria in the small intestine originate?

Traditional views hold that SIBO is closely associated with reduced intestinal motility, a weakened gastric acid barrier, and colonic bacterial reflux due to ileocecal valve dysfunction [13,15,16]. However, recent advances in microbiome research have provided important supplements and challenges to these traditional concepts (Table 1). Using quantitative metagenomic analysis, researchers have found that the overgrown microbiota in the duodenum of SIBO patients are predominantly Proteobacteria, such as Enterobacteriaceae and *Klebsiella*, rather than typical colonic anaerobes [17], and there is a clear genomic overlap between these bacteria and the oral microbiome [18]. This suggests that top-down oral microbial input may be another important pathway associated with SIBO. This raises a key scientific question: can intervention in the oral microbiome positively affect the prevention and treatment of SIBO by modulating the oral–gut axis?

**Table 1.** Comparison of traditional and modern microbiome perspectives on SIBO.

Feature	Traditional view (colonic retrograde migration)	Modern microbiome view
A major source of bacteria	Retrograde migration of colonic microbiota (Bacteroidetes, Firmicutes, etc.) [16]	Overgrowth of specific opportunistic pathogens (mainly Proteobacteria, e.g. *E. coli*, *K. pneumoniae*) [19]; oral microbiota (including the HACEK group, explicitly identified via paired saliva-duodenum tracking [18]^&^) as a potential top-down input source [18]
Core mechanism	Anatomical abnormalities (e.g. blind loop) or motility disorders leading to colonic reflux and colonisation [20]	Opportunistic overgrowth of specific species replacing the normal small‑intestinal microbiota; oral transmission, and gastric acid barrier weakening (e.g. PPI use) promote the process [16,21]
Microbial characteristics	Non‑specific increase in colonic anaerobes [16]	Significantly increased relative abundance of Proteobacteria (especially γ‑Proteobacteria, Enterobacteriaceae), decreased Firmicutes, reduced overall α‑diversity [19]
Association with symptoms	Related to malabsorption syndrome (diarrhoea, weight loss, malnutrition) due to total bacterial count increase [20]	Directly related to absolute or relative abundance of specific taxa/metabolites (e.g. Enterobacteriaceae with bloating, Aeromonadaceae with faecal urgency) [19]

^&^ The HACEK group enrichment was identified specifically within the subset of 21 paired saliva–duodenum samples analysed by Barlow et al., rather than as a cohort-wide finding across all 250 aspirates.

Abbreviations: PPIs, Proton Pump Inhibitors; HACEK: *Haemophilus* spp., *Aggregatibacter* spp., *Cardiobacterium* spp., *Eikenella* spp., and *Kingella* spp.

To maintain scientific clarity, a strict distinction must be made between established ecological observations and our speculative clinical hypotheses regarding disease pathogenesis. While existing literature firmly documents an oral–gut microbial overlap—demonstrating that oral microbiota can survive gastric passage and ectopically colonise the distal gastrointestinal tract—the functional consequences and clinical utility of oral hygiene remain unproven. To date, no human clinical trial has evaluated whether mechanical or chemical oral care can prevent, treat, or reduce the recurrence of SIBO. Current data are strictly limited to animal models, cross-sectional correlations, and proxy endpoints; thus, the use of oral interventions to disrupt microbial migration and alter SIBO risk remains a novel hypothesis requiring validation through rigorous prospective trials.

Accordingly, this narrative review aims to synthesise the current literature on the oral–gut axis and explore how oral dysbiosis may contribute to the pathogenesis of SIBO. Crucially, this manuscript represents a hypothesis-generating review designed to highlight potential interconnected biological pathways and propose future directions for clinical trials; it should not be interpreted as evidence-based clinical guidance for current practice. By framing these potential preventive opportunities through oral care, we hope to stimulate rigorous empirical testing to validate the proposed relationships and provide a theoretical framework for exploring interdisciplinary, microbiota‑targeted strategies for managing intestinal diseases.

## Clinical background of SIBO and evidence linking it to the oral–gut axis

### SIBO: definition, diagnostic dilemmas, and current treatment

SIBO is defined as an abnormal increase in bacterial quantity or alteration in the composition of the small intestinal microbiota [13]. The gold standard for diagnosis is culture of proximal small intestinal aspirate [22,23]. Traditionally, a count of ≥10^5^ CFU/mL in the jejunal aspirate was considered diagnostic of SIBO, but this threshold is usually restricted to specific anatomical conditions, such as blind loop syndrome [22,23]. The North American consensus and the American College of Gastroenterology (ACG) clinical guideline updated the diagnostic threshold to ≥10^3^ CFU/mL [22,23]. Small bowel aspirate and culture is invasive and technically demanding, limiting its widespread use; therefore, non‑invasive hydrogen/methane breath tests are commonly used in clinical practice. However, breath test results can be heavily influenced by intestinal transit time, background microbiota, colonic fermentation, and other factors, and their diagnostic accuracy remains controversial [20,24].

Furthermore, the diagnostic validity of breath-test-defined SIBO as an independent, primary clinical entity remains highly contested within modern gastroenterology. As outlined in the joint European Society of Neurogastroenterology and Motility (ESNM) and the American Neurogastroenterology and Motility Society (ANMS) clinical practice update, positive results on non-invasive hydrogen and methane breath tests frequently reflect physiological variations in background colonic fermentation or accelerated intestinal transit times, rather than true small bowel overgrowth [24]. Sceptics note that the overrepresentation of specific taxa in the proximal small intestine may reflect a secondary symptom of underlying functional dyspepsia, visceral hypersensitivity, or delayed gastric motility, rather than a causal driver of gastrointestinal pathology. Consequently, duodenal microbial signatures must be interpreted with caution, as null findings regarding true bacterial overgrowth are common when rigorous, direct aspirate cultures are utilised instead of breath testing [24].

Treatment of suspected SIBO primarily relies on empirical antibiotics [25]. Rifaximin, a minimally absorbed antibiotic, is the most studied and recommended first‑line therapy, with alternatives such as metronidazole and ciprofloxacin [25]. A landmark study tracking 80 SIBO patients by Lauritano et al [26]. found that after successful initial eradication with rifaximin (1200 mg/day for 7 days), the 6‑month recurrence rate, as detected by glucose breath test, was exactly 27.5%, with recurrence rates continuing to increase over time [26]. Oral probiotics, dietary adjustments, and reduction of proton pump inhibitor (PPI) use can be adjunctive measures, but their efficacy varies [13,25]. The limitations of diagnostic methods, the fundamental clinical scepticism surrounding breath testing, and the high recurrence rate after treatment highlight the need for standardised diagnostic procedures and novel, cautious preventive and therapeutic strategies.

### Evidence for the oral cavity as a potential source of SIBO‑associated bacteria

The oropharynx is the beginning of the digestive tract. Physiologically, approximately 1.5 liters of saliva are swallowed into the gastrointestinal tract each day, representing the most direct route for oral microbial migration to the gut [27]. Under healthy conditions, the body possesses multiple defence mechanisms to maintain a low bacterial load and a stable microbial structure in the upper digestive tract, especially the small intestine. These mechanisms include gastric acid, digestive enzymes, intestinal motility, the intestinal mucus layer, immunoglobulin A (IgA), and the “colonisation resistance” provided by the resident gut microbiota [28]. However, when these defence barriers are weakened (e.g. by reduced gastric acid or motility disorders) or when the invasive capacity of pathogenic bacteria is enhanced, gut dysbiosis can occur.

Multiple lines of evidence support the potential involvement of oral microbiota in SIBO pathogenesis (Table 2). Compelling preliminary evidence comes from the quantitative sequencing study by Barlow et al. [18], which analysed duodenal aspirates in a cohort of 250 adults and revealed that specific duodenal “disruptor taxa” (such as *Klebsiella* and *E. coli*) positively correlated with both SIBO and symptom severity [18]. Notably, while the absolute-load and symptom correlation analyses were conducted across this larger cohort, the genomic evidence linking these “disruptor taxa” to an oral origin relies on a smaller subset of 21 paired saliva–duodenum samples [18]. Within this paired sub-cohort, these specific strains exhibited unique genomic features indicative of an oral origin [18]. While these genomic overlaps offer a compelling mechanism for top–down oral–gut translocation, they represent localised findings that warrant further validation in larger clinical cohorts. Additionally, studies have shown that patients with functional dyspepsia exhibit increased abundance of Spirochaetes and decreased abundance of Fusobacteria and Saccharibacteria (TM7) in their oral microbiota, along with a higher Community Periodontal Index (CPI) and a higher prevalence of SIBO compared with healthy controls [29]. A higher SIBO positivity rate has also been observed in patients with recurrent aphthous ulcers [30]. These findings suggest that oral dysbiosis and oral conditions correlate with increased susceptibility to SIBO through complex systemic pathways.

**Table 2.** Microbiological evidence supporting oral microbiota involvement in SIBO pathogenesis.

Authors	Design/sample	Key findings	Implications for SIBO pathogenesis	Limitations
Barlow et al.	Cross‑sectional; quantitative sequencing of duodenal aspirates from 250 adults^#^	Identified oral‑derived “disruptor taxa” (e.g. *Klebsiella*, Enterobacteriaceae) associated with SIBO; their abundance positively correlated with symptom severity [18]	Clinical correlation demonstrating that duodenal “disruptor taxa” overlap genomically with bacteria of oral origin, supports the plausibility of top-down microbial translocation.	A cross-sectional design cannot establish causality.
Bao J et al.	Experimental; transplantation of saliva from periodontitis patients into germ‑free mice	Salivary microbiota colonised the mouse gut, induced gut dysbiosis, and triggered Th1‑mediated intestinal inflammation [31]	Experimental evidence that oral microbes can survive in the gut and affect intestinal ecology	Animal model; differences from human chronic disease settings
Liu XJ et al.	Case‑control; 21 FD patients vs. 12 healthy controls	Salivary microbiota composition, salivary pH, and CPI differed between FD patients and controls; SIBO proportion was higher in the FD group [29]	Clinical association between oral dysbiosis and SIBO susceptibility	Small sample size; FD and SIBO diagnoses based on symptoms and breath tests
Zhu J et al.	Prospective cohort; healthy volunteers using PPIs	PPI use significantly increased oral‑to‑gut microbial transmission, altering gut microbiota composition (e.g. increased *Streptococcus*) [21]	Gastric acid barrier disruption amplifies consequences of oral microbial migration, a key mechanism for PPI‑associated SIBO risk	SIBO endpoint not directly assessed

^#^ N = 250 for duodenal aspirate load; subset of N = 21 paired saliva–duodenum samples for oral-origin translocation data.

Furthermore, studies of molecular mechanisms have revealed how oral pathogens translocate into the gut and interact with tissue environments. Classic periodontal pathogens such as *Fusobacterium nucleatum* and *Porphyromonas gingivalis (P. gingivalis)* have been shown to colonise the gut through various survival strategies ectopically [28]. For instance, certain oral‑derived *Klebsiella* strains possess a specific gene cluster for adhesion in the inflamed gut, enabling them to adapt to and colonise this environment [32]. Once colonised, these bacteria can interact with the intestinal barrier by inducing ferroptosis in intestinal epithelial cells [33], influence the Th17/Treg immune balance, and alter short‑chain fatty acid (SCFA) metabolism, thereby correlating with local inflammation and creating a favourable environment for the initiation and persistence of SIBO [27,34,35].

Animal experiments have confirmed that oral administration of *P. gingivalis* alone correlates with gut dysbiosis and intestinal barrier dysfunction, thereby promoting intestinal inflammation [36,37]. Transplantation of saliva from periodontitis patients also induces gut alterations and low‑grade inflammation in recipient germ-free mice [31]. Long‑term PPI use weakens the gastric acid barrier and significantly increases oral‑to‑gut bacterial transmission, altering the gut microbiota [15]. These lines of evidence suggest that oral dysbiosis may increase the input of intestinal pathogens and thereby correlate with SIBO, although causality remains unproven (Figure 1).

**Figure 1.**
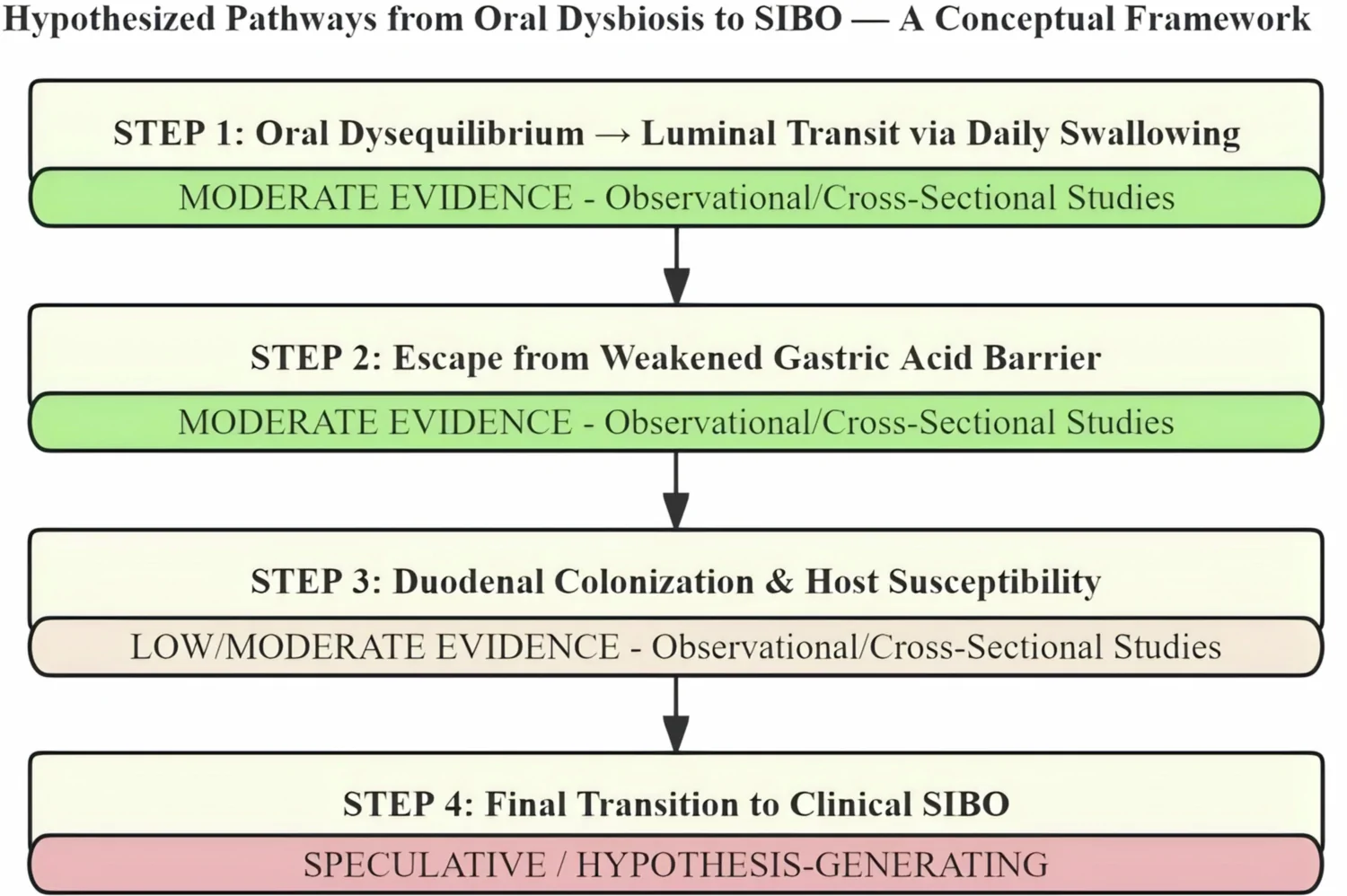
Hypothesised mechanisms by which oral microbiota dysbiosis may contribute to SIBO. Oral dysbiosis may lead to the overgrowth of potentially pathogenic bacteria (e.g. Klebsiella, Enterobacteriaceae). These microbes and their products (inflammatory cytokines, LPS, SCFAs) may reach the small intestine via swallowing. In individuals with predisposing factors (e.g. reduced gastric acid, impaired motility), such exposures may compromise intestinal barrier function and immune homoeostasis, potentially facilitating small intestinal colonisation and overgrowth (SIBO). This figure illustrates a hypothesised mechanism; direct evidence for causal links remains limited.

Based on the cumulative evidence, we propose a unified, testable hypothesis: effective oral hygiene practices that reduce the overall load of oral “disruptor bacteria” can decrease their continuous downstream input into the small intestine, thereby helping to prevent initial SIBO development, assist in acute treatment, and reduce long-term clinical recurrence rates.

## Modulation of the oral microbiome by oral hygiene measures and potential downstream effects

Oral hygiene is a fundamental practice for maintaining oral health, primarily involving mechanical methods (e.g. toothbrushing, flossing, or water flossing) and chemical methods (e.g. mouthwashes and toothpastes with active ingredients). Its core function is to regulate the complex oral biofilm, and the effects of different interventions vary (Table 3).

**Table 3.** Effects of different types of oral hygiene interventions on the oral and systemic microbiome.

Intervention type	Representative ingredients/methods	Main effects on the oral microbiome	Potential impact on the oral–gut axis	Level of Evidence (Oral vs. Gut Impact)
Mechanical cleaning	Toothbrushing, water flossing	Reduces plaque biomass, alters microbial composition [38,39]	By disrupting oral biofilms and removing plaque/biofilm debris [38,39], it may reduce the microbial load available for swallowing; However, there is currently a lack of direct measurement of swallowing microbial load	**Oral:** High (RCTs) **Gut:** Mechanistic hypothesis/Theoretical
Broad‑spectrum chemical antimicrobial	Chlorhexidine (CHX)	Potent, non‑selective antimicrobial; long‑term use causes dysbiosis and reduced diversity [40]	Hypothesised to alter the composition of descending microbiota and disrupt the protective nitrate–NO pathway. Lacks direct clinical evidence regarding SIBO outcomes; long-term safety is unverified and requires cautious evaluation rather than clinical implementation [40]	**Oral:** High (RCTs/Observational) **Gut:** Preclinical (Animal models [40])/Mechanistic hypothesis
Selective/natural chemical agents	Essential oils, propolis, coconut oil, enzymes, Sn^2^⁺	Inhibit specific pathogens with minimal impact on overall microbial balance, or help maintain ecological stability [41–45]	May precisely reduce oral colonisation of interfering bacteria (e.g. Enterobacteriaceae), thus reducing their gut input. However, efficacy against specific interfering bacteria and long‑term safety need verification	**Oral:** Moderate to High (RCTs [41–44]) **Gut:** Mechanistic hypothesis/Theoretical
Functional ingredients	Specific probiotics	Competitive exclusion of pathogens, modulation of local immunity, promotion of healthy microbiota [46]	Maintains oral homoeostasis via enhancing resistance rather than simple sterilisation; may have more positive indirect effects on the gut	**Oral:** Moderate to High (RCTs [46]) **Gut:** Mechanistic hypothesis/Theoretical

### Mechanical cleaning

Mechanical cleaning effectively reduces plaque biomass and thus lowers the overall oral microbial load. For example, in a randomised controlled trial evaluating the adjunctive effect of water flossing, after 12 weeks of continuous use, participants' supragingival plaque microbial composition changed significantly: the abundance of *Prevotella* (an anaerobic genus) decreased markedly [38]. This suggests that water flossing may disrupt the biofilm structure through physical flushing, thereby improving the local oxygen environment and promoting a shift toward aerobic microbiota, ultimately alleviating gingival inflammation and halitosis [38,47]. These effects demonstrate the ability of mechanical cleaning to control plaque quantity and reveal its potential role in modulating microbial ecological function, indicating that adjunctive tools like water flossing have positive clinical significance for optimising the oral microbiome.

On the other hand, for individuals with a complex oral environment, inadequate mechanical cleaning can easily lead to dysbiosis and an increase in potentially pathogenic bacteria [48]. Studies have found that during fixed orthodontic treatment, even though patients increase their toothbrushing frequency, the presence of appliances significantly disrupts the composition and diversity of the oral microbiota [48]. For example, brackets and bands create niches for plaque retention, leading to increased microbial diversity and potentially promoting the abundance of potentially pathogenic phyla such as Firmicutes and Spirochaetes [48]. Firmicutes include many acidogenic and cariogenic bacteria (e.g. *Streptococcus*), and their increase can elevate the risk of dental caries [1]. At the same time, some Spirochaete species are closely associated with periodontal inflammation, and their increased abundance may exacerbate gingivitis or even destroy periodontal tissues [49].

Thus, in the specific condition of wearing orthodontic appliances, conventional toothbrushing alone is often insufficient to remove plaque effectively [48], and it is necessary to intensify mechanical cleaning, for example, by combining floss or water flossing, to limit the overgrowth of pathogenic microorganisms and maintain oral ecosystem stability. Given that oral microorganisms can enter the digestive tract through swallowing [27], orthodontic treatment‑induced changes in the oral microbiome could theoretically correlate with gut microbiota balance and be potentially linked to SIBO. This suggests that for individuals at high risk of SIBO, ensuring effective mechanical cleaning is a primary line of defence for maintaining oral microbial balance.

### Chemical cleaning agents

Chemical cleaning agents exert their oral care effects mainly through antibacterial and anti‑inflammatory mechanisms. Traditional broad‑spectrum antibacterial mouthwashes, such as chlorhexidine (CHX), have been proven to effectively reduce dental plaque, but their action lacks selectivity [50,51]. Long‑term use of such products not only leads to reduced oral microbial diversity and emergence of resistant strains, but also significantly inhibits oral nitrate‑reducing bacteria, thereby interfering with the nitrate–nitrite–nitric oxide (NO) metabolic pathway, which is crucial for cardiovascular and gastrointestinal mucosal health [50,52].

More notably, the use of antibacterial mouthwashes (e.g. CHX) may indirectly affect the body systemically by modulating the oral–gut axis. Animal experiments in Western-diet-fed mice demonstrate that long‑term CHX exposure can alter the gut microbiota structure sufficiently to disrupt normal nutrient absorption [40]. In a mouse model of obesity induced by a high‑fat, high‑sugar, low‑fibre Western diet, CHX oral care reduced gut microbial richness and significantly altered microbial composition [40]. Since some gut bacteria play important roles in bile acid and steroid metabolism [53], these changes could interfere with fat and protein absorption, ultimately manifesting as a pathological disruption of digestion marked by slower weight gain, reduced body fat and liver fat, and lower plasma insulin levels [40]. Rather than demonstrating a therapeutic benefit, these findings highlight that non-selective chemical ablation of the oral microbiota can induce unexpected systemic alterations along the distal gastrointestinal tract that mirror malabsorption states [40]. Additionally, although not evaluating a direct SIBO clinical endpoint, clinical trials that track proxy sites along the upper digestive tract have found that antibacterial mouthwash use alters the composition of the oesophageal microbiota and its tissue gene expression patterns [54]. Together, these studies indicate that chemical interventions targeting the oral cavity can have effects that extend to the distal digestive tract.

Notably, the above findings lead to an important, hypothesis-generating clinical inference: the use of broad‑spectrum antibacterial mouthwashes may, in certain contexts, paradoxically correlate with an increased susceptibility to SIBO. The potential mechanisms mainly involve two pathways. First, non‑selective antibacterial agents such as CHX significantly inhibit oral nitrate‑reducing bacteria (e.g. *Actinomyces, Veillonella*), thereby interfering with the nitrate→nitrite→NO conversion pathway [50,55]. NO plays a critical role in maintaining small intestinal smooth muscle motility, mucosal blood flow, and epithelial barrier integrity [52]; reduced NO production may weaken the small intestine's ability to physically clear bacteria, creating conditions for bacterial overgrowth [52,56]. Second, long‑term use of broad‑spectrum antibacterial mouthwashes can reduce oral microbial diversity, paradoxically promoting the overgrowth of some opportunistic pathogens (e.g. Enterobacteriaceae) or resistant strains [50,51]. These bacteria are swallowed in large numbers daily [57]; if an individual also has a weakened gastric acid barrier (e.g. long‑term PPI use) or small-intestinal motility disorders, they can easily colonise and overgrow in the small intestine, potentially contributing to the development or recurrence of SIBO [13,15,16]. It should be emphasised that the above inferences are currently based mainly on mechanistic studies and animal experiments [40], and there is an absolute lack of prospective clinical studies directly evaluating the relationship between long‑term broad‑spectrum antibacterial mouthwash use and definitive SIBO endpoints.

### Novel and targeted products

The current development concept of oral care products is shifting from broad‑spectrum sterilisation to targeted modulation, aiming to maintain or restore the balance of the oral microbiome [51,58,59]. Relevant active ingredients include certain plant essential oils (e.g. tea tree oil, thymol), propolis extracts, enzyme preparations (e.g. glucose oxidase, lactoperoxidase), and metal ions (e.g. Sn^2^⁺) [43–45,59–61]. These components are designed to more precisely inhibit periodontal pathogens while having less impact on commensal bacteria, and may even support the growth of health‑associated bacteria [59]. From the perspective of the oral–gut axis, such products could theoretically reduce the total number of interfering bacteria entering the gut while retaining bacteria beneficial to the mouth, although direct evidence for this top-down effect remains lacking. This targeted strategy may offer a more biologically balanced alternative to broad-spectrum chemical agents, avoiding disruptions to metabolic and protective pathways hypothesised to affect the distal digestive tract.

## Potential mechanisms by which oral hygiene interventions may affect SIBO

Based on current, highly indirect evidence, we propose that oral hygiene might theoretically influence the occurrence and development of breath-test-defined SIBO through the following interconnected mechanisms (Figure 2). It must be explicitly emphasised that these proposed pathways are derived from heterogeneous, often non-SIBO clinical proxy models or animal experiments; none of these mechanisms have been directly verified or clinically validated in human SIBO cohorts.

**Figure 2.**
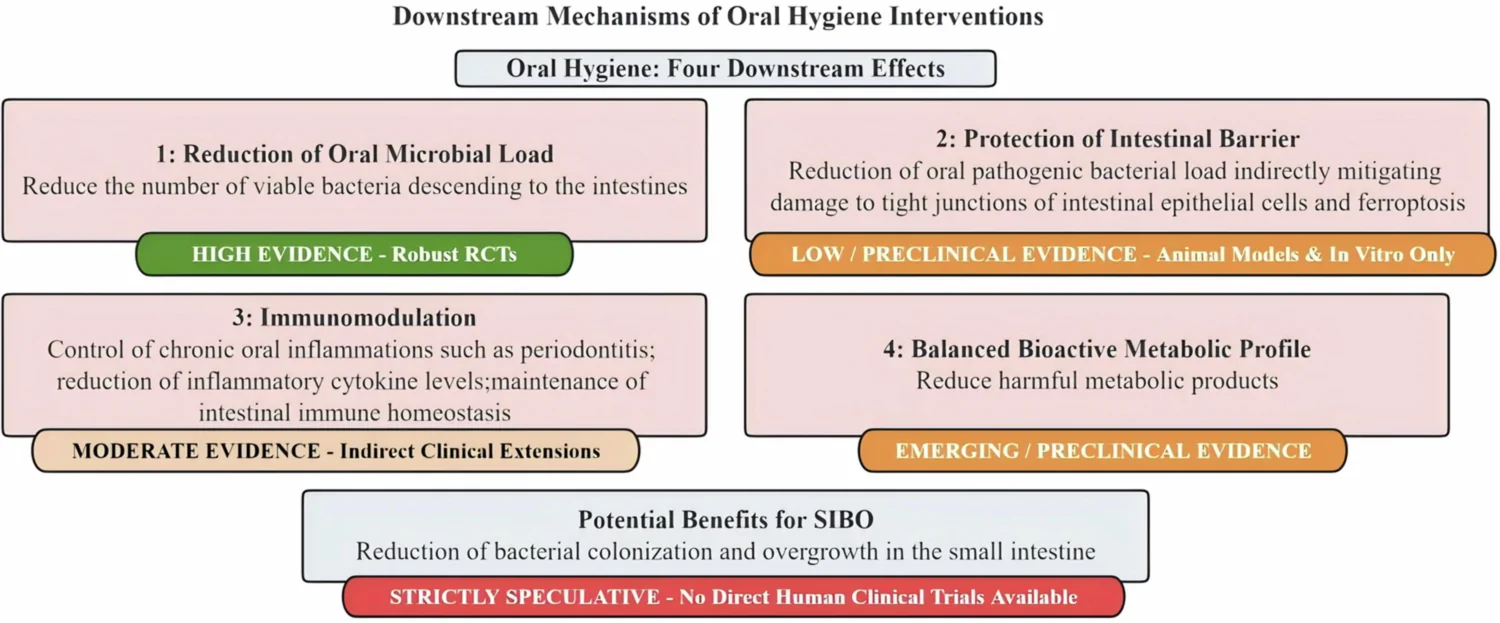
Hypothesised mechanisms by which oral hygiene may intervene in SIBO. This figure illustrates four interconnected pathways through which oral hygiene may reduce SIBO risk: (1) reducing oral microbial load, decreasing viable bacteria descending to the intestine; (2) protecting the intestinal barrier by mitigating pathogen‑induced ferroptosis and tight junction disruption; (3) modulating systemic and gut‑local immunity by lowering inflammatory cytokines and maintaining immune homoeostasis; (4) balancing bioactive metabolite profiles to reduce harmful products. These pathways converge to decrease small intestinal colonisation and overgrowth of disruptor taxa, thereby lowering SIBO risk or recurrence, but direct clinical evidence for this hypothesis is currently lacking.

### Reducing the hypothesised oral microbial load

SIBO‑associated “disruptor taxa” (e.g. Enterobacteriaceae) are commonly found in already dysbiotic oral environments [18,57,62]. Effective oral hygiene measures, especially the use of targeted oral care products that inhibit these bacteria, could theoretically reduce their colonisation in the oropharynx [59]. During daily swallowing, the number of pathogens entering the gut would correspondingly decrease, potentially reducing the likelihood that these bacteria successfully colonise and overgrow in contexts where the gastric acid barrier is weakened or local intestinal defences are compromised [18,57]. If, in future interventional studies, subjects receiving a specific oral hygiene method show a greater reduction in the absolute abundance of interfering bacteria in saliva or subgingival plaque compared to a placebo group, and this reduction correlates with subsequent changes in the abundance of similar bacteria in the gut, it would provide valuable preliminary support for this top-down transmission model. However, high‑quality randomised controlled trials are needed to move this concept from a plausibility argument to a validated mechanism, as this pathway currently lacks direct clinical validation in human SIBO cohorts.

### Hypothetically protecting and regulating intestinal barrier function

Pre-clinical studies have confirmed that oral pathogens such as *P. gingivalis* and *F. nucleatum* can damage intestinal barrier function *in vitro* by inducing ferroptosis in intestinal epithelial cells and disrupting tight junction proteins [33,36]. Animal experiments have also shown that oral administration of *P. gingivalis* causes intestinal barrier dysfunction and increased intestinal permeability in rodent models [36]. Reducing the load of these pathogens in the oral cavity through targeted oral hygiene measures could theoretically alleviate their continuous, descending attack on the intestinal barrier, while also reducing the number of live bacteria and toxins swallowed into the gut [31,36]. Additionally, preclinical studies in rodent models provide evidence that bioactive mouthwashes containing inorganic nitrates can increase the relative abundance of nitrate-reducing bacteria in the oral cavity and promote the nitrate-nitrite-NO metabolic pathway [63], which is known to play a critical role in maintaining gastrointestinal mucosal blood flow and barrier function [52]; these interventions also elevate circulating nitrate/nitrite levels and reduce blood pressure [63]. However, the chlorhexidine group showed no such changes. Nevertheless, translating these laboratory findings into human clinical practice remains speculative, and this pathway lacks direct clinical validation in human SIBO cohorts.

### Modulating systemic and gut‑local immunity

An associative relationship exists between oral dysbiosis and systemic low‑grade inflammation. Periodontitis, as a chronic infectious focus, can be associated with elevated levels of inflammatory cytokines (e.g. TNF‑α, IL‑1β, IL‑6) in the bloodstream [64,65]. It is hypothesised that a resulting systemic inflammatory state might disturb gut immune homoeostasis and alter the intestinal microenvironment, potentially making it more conducive to opportunistic pathogen colonisation [56]. Effective periodontal therapy and oral hygiene measures could reduce local periodontal inflammation and lower systemic inflammatory marker levels [66]. Whether this translates into an immune environment less conducive to SIBO, however, remains a theoretical possibility that lacks clinical validation. At the gut‑local level, certain bacteria of oral origin (e.g. *Klebsiella* isolated from the saliva of Crohn’s disease patients) have been shown to induce Th1‑type immune responses in mouse models [67]. Reducing oral exposure to such pro‑inflammatory bacteria might help maintain the balance between intestinal Th17 and Treg immune cells, which is crucial for preserving intestinal immune tolerance and preventing excessive inflammation [35]. Based on this framework, in SIBO patients with mild periodontitis, a reduction in serum inflammatory markers after standard periodontal treatment should theoretically correlate with the degree of relief of SIBO‑related symptoms. However, this clinical correlation remains entirely untested, and this immune-modulatory pathway lacks direct clinical validation in human SIBO cohorts.

### Influencing microbial and gut–distal organ metabolism

The metabolic functions of the oral microbiome are proposed to influence the composition of metabolites that enter the gut via swallowing, thereby potentially regulating the small intestinal microenvironment [52]. For example, dietary nitrate is reduced to nitrite primarily by oral nitrate-reducing bacteria [68]. After entering the stomach, nitrite is further converted to NO [68], which regulates gastrointestinal blood flow, motility, and mucosal defence [52]. However, the use of broad‑spectrum antibacterial mouthwashes is hypothesised to interfere with this conversion process [50]. Reduced NO production might weaken small intestinal motility and mucosal defence [52], theoretically creating conditions conducive to bacterial overgrowth. In addition, oral microbial dysbiosis can introduce inflammatory metabolites, such as lipopolysaccharide (LPS), into the upper gastrointestinal tract, potentially inducing secondary local dysbiosis [6,36]. Upon entering the small intestine, these communities and their byproducts may compromise mucosal barrier function and disrupt localised immune homoeostasis [6,36]. Therefore, while maintaining a healthy and stable oral microbiome is hypothesised to preserve NO‑mediated small intestinal defence functions and reduce the risk of overgrowth induced by abnormal input of microbial metabolites, these pathways are based on highly fragmented data. This metabolic link currently represents an unproven model that completely lacks direct clinical validation in human SIBO cohorts.

## Current limitations, controversies, and future directions

Despite significant progress in research on the oral–gut axis, several key limitations and controversies remain that need to be addressed in future studies. Using oral hygiene as a strategy for SIBO prevention and treatment also faces major evidence gaps and challenges.

### Major limitations and controversies

Current studies investigating the relationship between the oral–gut axis and SIBO face multiple methodological challenges. The vast majority of human studies use cross‑sectional or case‑control designs [18,29], which can only suggest associations between oral dysbiosis and intestinal clinical markers but cannot determine causality [69]. Second, the diagnosis of SIBO relies heavily on controversial hydrogen breath tests or demanding fluid cultures [13,22], which lack universally accepted cutoff values and cannot distinguish specific bacterial species [70]. In addition, sampling sites (e.g. saliva, plaque) and sample preservation methods for oral microbiota vary greatly across studies [71–73], making cross‑study comparisons difficult. Third, translating animal model findings to clinical settings is highly challenging. For example, oral gavage of pathogens in mice [36,37] cannot easily replicate the long‑term progression of chronic periodontitis or the complex immune environment in humans; thus, conclusions from animal experiments should be extrapolated to clinical practice with extreme caution.

Moreover, the long‑term risks of oral interventions remain unclear, and high‑quality evidence is lacking. A particularly noteworthy hypothesis-generating controversy is that broad‑spectrum antibacterial mouthwashes (e.g. CHX) may theoretically increase the risk of SIBO. As described in Section 3.2, the proposed mechanisms involve inhibition of the NO pathway and overgrowth of opportunistic pathogens. However, this hypothesis is currently supported only indirectly by mechanistic models and animal experiments that did not directly measure SIBO [40,52], and there is an absolute lack of high‑level clinical evidence directly evaluating a causal relationship between broad‑spectrum mouthwash use and SIBO incidence. Therefore, this represents both a major knowledge gap and a key controversy that prospective cohort studies or randomised controlled trials need to address before any clinical recommendations can be made safely. At the same time, the long‑term safety of novel selective products (including plant extracts, metal ions, enzymes, etc.) and their true impact on SIBO risk remain to be verified [44,59,60,74].

More critically, there is a severe lack of high‑level clinical evidence directly assessing the effect of oral hygiene interventions on SIBO outcomes, and study designs often fail to adequately control for major baseline confounders. Beyond PPI use and dietary patterns, factors such as tobacco smoking and antibiotic exposure must be strictly controlled or stratified, as both can significantly alter microbial communities in the oral cavity and gastrointestinal tract [6,20,75,76], albeit with differing temporal dynamics and magnitudes of effect [77]. Specifically, chronic tobacco use has been associated with shifts in the oral microbial community, including alterations in local oxygen tension [78], and may compromise upper-gastrointestinal mucosal defence [79]. On the other hand, systemic antibiotic exposure can disrupt local ecological resilience and select for opportunistic taxa [80,81], thereby altering microbial metabolic functions [82] and potentially contributing to the pathophysiology of SIBO.

Finally, while our model focuses on the anterograde (top-down) migration of oral pathogens, the oral–gut axis is inherently bidirectional, introducing a retrograde feedback loop that could obscure direct causal mechanisms [83]. Chronic intestinal dysbiosis and the metabolic byproducts of SIBO can trigger low-grade systemic inflammation, elevating pro-inflammatory cytokines that compromise the periodontal vascular bed and alter salivary kinetics [83]. Furthermore, massive luminal gas production in SIBO can modify the microenvironment of the upper digestive tract, potentially shaping oral niche characteristics and creating a self-sustaining pathological cycle in which oral dysbiosis seeds the gut, and the resulting overgrowth reinforces oral dysbiosis [84].

### Future research directions

Based on the above limitations, future research should focus on several key directions to move the oral–gut axis from theoretical association to mechanistic validation and clinical translation. The most urgent need is to fill the evidence gap between oral hygiene interventions and SIBO clinical outcomes.

Accordingly, we propose the following priorities for future research:

(1) **Hypothesis‑testing clinical trials**: Design randomised controlled trials with SIBO clinical endpoints (e.g. breath test normalisation rate, symptom relief, and recurrence rates) in high‑risk populations (e.g. long‑term PPI users and patients with functional dyspepsia), to compare whether specific oral hygiene methods reduce SIBO‑associated interfering bacteria in saliva compared to usual care. To ensure that variations in SIBO risk are attributable to oral care rather than to systemic microbial disruptors, these trials must implement rigorous antibiotic washout periods (e.g. 4–12 weeks) and meticulously account for smoking status.
(2) **Bidirectional Feedback and Chronological Sequence Testing:** To isolate top-down mechanisms and prevent retrograde confounding, future clinical trials must utilise longitudinal, high-frequency sampling of both saliva and duodenal aspirates or faeces. Tracking the precise chronological sequence of these microbial shifts is essential to determine whether longitudinal changes in oral interfering bacteria genuinely precede clinical SIBO onset, or if concurrent eradication of the intestinal overgrowth is required to break the loop and stabilise the oral ecosystem.
(3) **Mechanistic studies**: Use metagenomic and metabolomic techniques to track the transmission trajectory of “interfering bacteria” from the oral cavity to the small intestine and their metabolic functional changes before and after oral hygiene interventions.
(4) **Safety assessment**: Systematically evaluate the true impact of long‑term use of different types of oral hygiene products on the oral–gut axis and overall health, clarifying the balance between benefits and risks.

## Conclusion

Oral dysbiosis is no longer viewed merely as a localised pathological state but as a potential upstream factor closely linked to downstream alterations in the gut via the oral–gut axis. Available evidence indicates that orally derived microorganisms (e.g. *Klebsiella,* Enterobacteriaceae), inflammatory mediators, and aberrant metabolites are continuously delivered into the digestive tract through daily swallowing, and in concert with host factors such as a weakened gastric acid barrier and small intestinal dysmotility, they may potentially contribute to the development or maintenance of SIBO. Therefore, oral dysbiosis has the potential to represent a modifiable risk factor for SIBO, although this remains to be validated in prospective clinical studies.

Viewing SIBO through the lens of the top-down oral–gut axis hypothesis shifts our clinical perspective away from traditional colonic reflux models. Testing this conceptual framework would require a coordinated shift in research paradigms toward evaluating upstream interventions, focusing on targeted oral hygiene interventions to reduce the descending pathobiont load and protect the vulnerable small intestinal microenvironment. Among various oral hygiene strategies, mechanical cleaning (e.g. toothbrushing combined with water flossing) can effectively reduce plaque biomass. The impact of chemical cleaning agents is more complex. While broad‑spectrum antibacterial mouthwashes (e.g. CHX) can control plaque in the short term, their non‑selective antimicrobial action may disrupt the nitrate‑NO protective pathway and induce overgrowth of opportunistic pathogens, theoretically posing a risk of backfiring in SIBO‑prone populations. In contrast, selective, eco‑friendly interventions (e.g. formulations containing stannous ions, plant essential oils, or enzymes) may theoretically reduce the load of interfering bacteria descending into the gut by modulating the oral microbiome; however, direct evidence for this downstream effect remains limited and is largely based on mechanistic reasoning.

However, it must be clearly stated that the above hypothesis is currently based primarily on microbiome association analyses, mechanistic studies, and animal experiments. Direct clinical evidence from high‑quality randomised controlled trials that establish oral hygiene as an effective preventive or therapeutic measure for SIBO remains lacking. This evidence gap is both the core limitation of current research and the primary obstacle to future translation.

In summary, oral hygiene, as a low‑cost, highly accessible daily practice, deserves serious exploration for its potential in SIBO management. Only through interdisciplinary, translational research that integrates oral microbiome modulation into the comprehensive prevention and treatment framework of digestive diseases can we objectively evaluate its true value and offer a new perspective for solving this clinical challenge.

## Data Availability

Data sharing is not applicable to this article as no new data were created or analysed in this study.

## Generative artificial intelligence

No generative artificial intelligence or AI‑assisted technologies were used in the preparation of this work.
